# Excessive IL-15 promotes cytotoxic CD4 + CD28− T cell-mediated renal injury in lupus nephritis

**DOI:** 10.1186/s12979-022-00305-9

**Published:** 2022-11-01

**Authors:** Ti Zhang, Xin Liu, Yue Zhao, Xiaodong Xu, Yaoyang Liu, Xin Wu

**Affiliations:** 1 Jinling Hospital, National Clinical Research Center of Kidney Diseases, Nanjing University School of Medicine, Nanjing, China; 2 Department of Rheumatology and Immunology, Shanghai Changzheng Hospital, The Second Military Medical University, Shanghai, China

**Keywords:** Lupus, Lupus nephritis, CD4 + CD28− T cell, IL-15

## Abstract

**Background:**

Patients with systemic lupus erythematosus (SLE) are highly susceptible to infection and cardiovascular events, suggesting that chronic antigenic stimulation may accelerate premature aging in SLE patients. Premature aging in SLE is often accompanied with the expansion of cytotoxic CD4 + CD28−T cells. Damage caused by CD4 + CD28− T cells enhances the progressive aging of the tissue function and loss of organism’s fitness. The high serum level of IL-15 has been implicated in the pathogenesis of SLE, but its role in CD4 + CD28−T cell-mediated cytotoxicity in nephritic SLE remains unclear. The aim of this study was to investigate the effect of IL-15 on functional properties and associated renal damage of cytotoxic CD4 + CD28− T cell in lupus nephritis (LN).

**Results:**

Flow cytometry showed that the number of circulating innate-like CD4 + CD28− T cells was increased in patients with nephritic SLE. Immunofluorescence showed CD4 + CD28− T cell infiltration in the kidney of LN patients, which was correlated with multiple clinicopathological features including estimated glomerular filtration rate (eGFR), proteinuria, the proportion of glomerulosclerosis and the degree of renal chronicity. In addition, a high level of IL-15 and IL15-expressing macrophage infiltration was detected in the periglomerular and intraglomerular tissues of LN patients, which enhanced the innate features, cytokine secretion and migratory capability of CD4 + CD28− T cells, and finally exerted direct TCR-independent cytotoxicity on glomerular endothelial cells in an IL-15-dependent manner in vitro.

**Conclusion:**

Our study demonstrated that excessive IL-15 potentially promoted cytotoxic CD4 + CD28− T cell-mediated renal damage in LN. This finding may provide new insights into the potential association of premature aging and tissue damage in LN.

**Supplementary information:**

The online version contains supplementary material available at 10.1186/s12979-022-00305-9.

## Introduction

Aging of the immune system or immunosenescence is a natural physiological process of aging, which increases susceptibility of individuals to chronic pathologies, including autoimmune diseases, infections and cancers, especially in the elderly population. Besides the increased serum levels of autoantibodies and pro-inflammatory cytokines, T lymphocyte aging is also a hallmark of immunosenescence characterized by the decreasing numbers of naïve T cells and the increased number of memory T cells and CD28 negative T cells [1, 2].

Systemic lupus erythematosus (SLE) is a highly complex and heterogeneous autoimmune disease characterized by chronic inflammation of multiple tissues and organs, including the skin, kidneys, and joints [3, 4]. Like aged individuals, SLE patients are susceptible to infections and cardiovascular diseases (CVD) [5]. Shortened telomere length and CD4 + CD28−T cell expansion were both observed at cellular and molecular levels in SLE patients [6, 7], leading to the hypothesis that repeated autoantigenic stimulation may be able to accelerate the immunosenescence process and related tissue damage in this population.

CD4 + C28−T cells accumulate in the circulation gradually with age increasing. In healthy people older than 65 years, CD4 + CD28− T cells generally account up to 50% of total CD4 + T cells, while only 0.1-2.5% CD4 + CD28− T cells of all CD4 + T cells are seen in young healthy individuals [8–10]. However, the inappropriate expansion of CD4 + C28−T cells was observed in many chronic inflammatory diseases, including autoimmune disorders such as SLE, rheumatoid arthritis and multiple sclerosis, and infectious diseases like infections with cytomegalovirus (CMV) [11–13]. Therefore, it is suggested that CD4 + CD28 − T cells may be a biomarker for premature immunosenescence under pathological conditions [14]. In addition, CD4 + CD28− T cells promote inflammation by producing high levels of inflammatory cytokines including tumor necrosis factor-α (TNF-α) and interferon-γ (IFN-γ), and releasing cytotoxic molecules including perforin and granzyme B which could damage the vascular wall in patients with acute coronary syndrome, which may provide an association between the high prevalence of CVD and SLE [15]. The damage caused by CD4 + CD28− T cells will also enhance the progressive aging in tissue function over time, which contribute to the loss of homeostasis and loss of the organism’s fitness ultimately. However, the underlying mechanism of subsequent renal tissue damage caused by cytotoxic CD4 + CD28− T cells in SLE with nephritis remains unknown.

IL-15 is specifically produced by monocytes, macrophages, and dendritic cells. IL-15 production could be induced by bacterial and viral infections associated with innate signals such as type I IFN, double-stranded RNA and TLR signaling [16]. Meanwhile, IL-15 could regulate tissue-resident T cells and limit tissue destruction, while chronically dysregulated IL-15 also promotes organ-specific autoimmune diseases including rheumatoid arthritis, multiple sclerosis, type 1 diabetes and celiac disease [17]. In multiple sclerosis, the potential pathogenic characteristics of CD4 + CD28− T cells including cytotoxic and proliferation could be augmented by IL-15 [18]. A higher serum level of IL-15 has also been implicated in the pathogenesis of SLE in some studies [19, 20], but the sample size was too small to draw a convincing conclusion. In addition, the role of IL-15 has not been fully evaluated in local renal damage in lupus nephritis (LN). Thus, the impact of IL-15 on functional properties of cytotoxic CD4 + CD28− T cells and their associated renal impairment are worthy of investigation.

The aim of the present study was to investigate the effect of IL-15 on cytotoxic CD4 + CD28− T cell activities in LN patients, describe the population and properties of CD4 + CD28− T cells in LN, and determine whether circulating and local IL-15 produced by macrophages could provide excess IL-15 to CD4 + CD28− T cells, thus producing the tissue-infiltrating and cytotoxic effects leading to potential renal impairment in vivo.

## Results

### CD4 + CD28− T cells are expanded in the circulation of SLE patients with nephritis

**Fig. 1:**
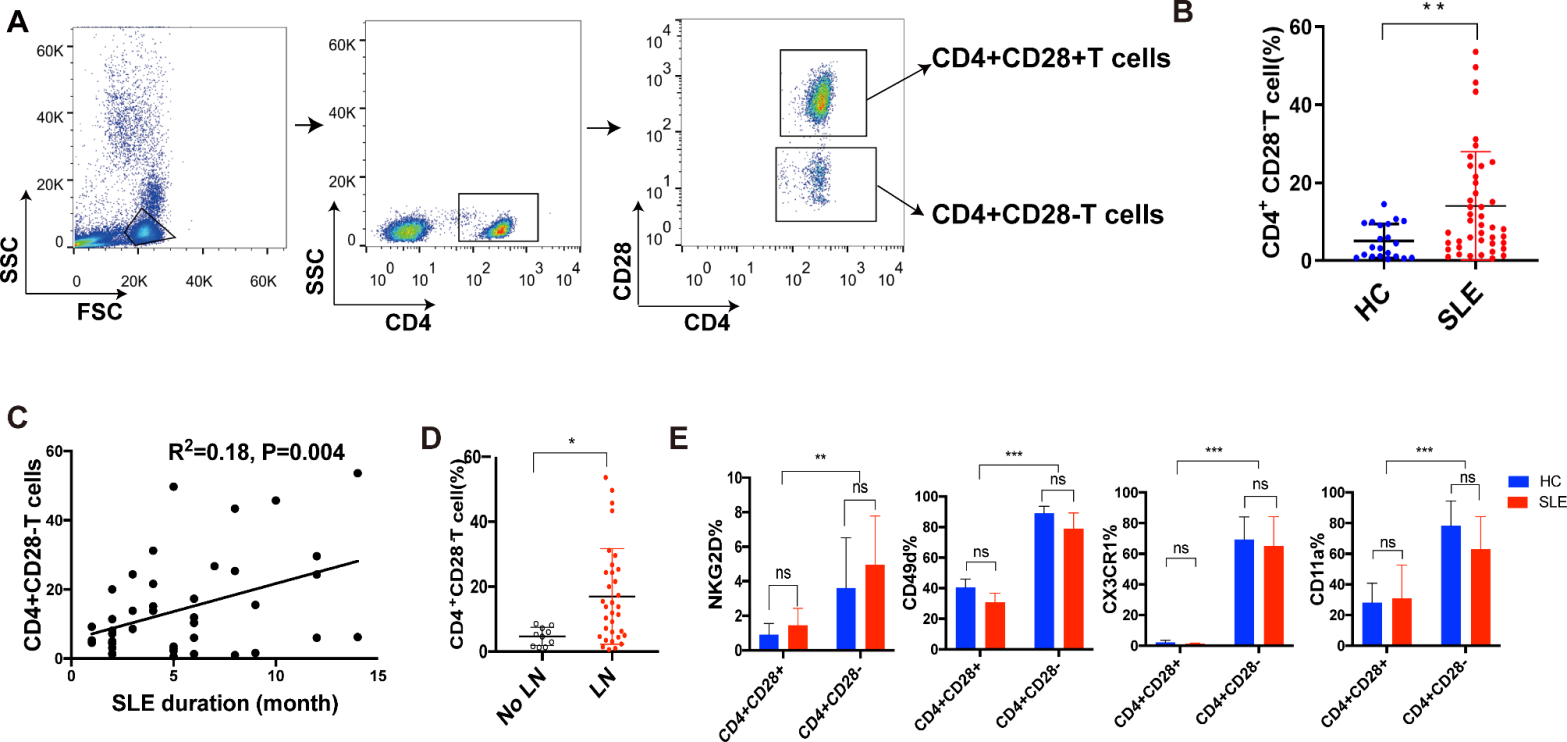
**Expanded CD4 + CD28− T cells in the circulation of SLE patients with nephritis exhibit innate-like function** (A) Representative flow cytometry dot plots showing the gating strategy used to define CD4 + CD28− T cells (B) A summary of the CD4 + CD28− T cell population in 21 healthy controls and 43 patients with SLE (5.03 ± 0.95% vs. 14.09 ± 2.12%, P = 0.0051). Each circle represents one individual. Horizontal bars indicate the mean (C) A correlation was found between the CD4 + CD28−T cell population and the SLE duration (D) The number of CD4 + CD28−T cells in 33 nephritic SLE patients was larger than that in 10 SLE patients without nephritis (E) Analysis of the expression levels of NKG2D and the chemokine receptor CX3CR1, integrin CD11a, CD49d on CD4 + CD28− and CD4 + CD28 + T cells from PBMCs of 10 healthy controls and 10 SLE patients. *P < 0.05; **P < 0.01; ***P < 0.001

Multiparameter flow cytometry showed that the percentage of CD4 + CD28− T cells in SLE patients was significantly higher than that in the age- and sex-matched healthy controls (14.09 ± 2.12% vs. 5.03 ± 0.95%, P = 0.0051) (Fig. 1A-B). There was a correlation between the SLE duration and the number of CD4 + CD28−T cells (Fig. 1C). In addition, the proportion of CD4 + CD28− T cells in SLE patients with nephritis was significantly higher than that in those without nephritis (16.94 ± 2.562 vs. 4.687 ± 0.88, P = 0.012) (Fig. 1D). No significant correlation was observed between the frequency of CD4 + CD28− T cells and the SLEDAI score. Also, there was no significant difference in the frequency of CD4 + CD28− T cells in treatment naive or treated patients **(Supplementary Figs.** 1–2).

The functional characteristics of CD4 + CD28− T cells in SLE were evaluated by flow cytometry. Compared with CD4 + CD28 + T cells, CD4 + CD28− T cells were equipped with innate-like functions, including highly expressed activating NK cell receptor NKG2D and migratory molecules including CD11a, CD49d and CX3CR1, which is consistent with the result previously reported (Fig. 1E).

### CD4 + CD28-T cell infiltration in the LN renal tissue is correlated with the clinicopathological characteristics of LN patients

**Fig. 2:**
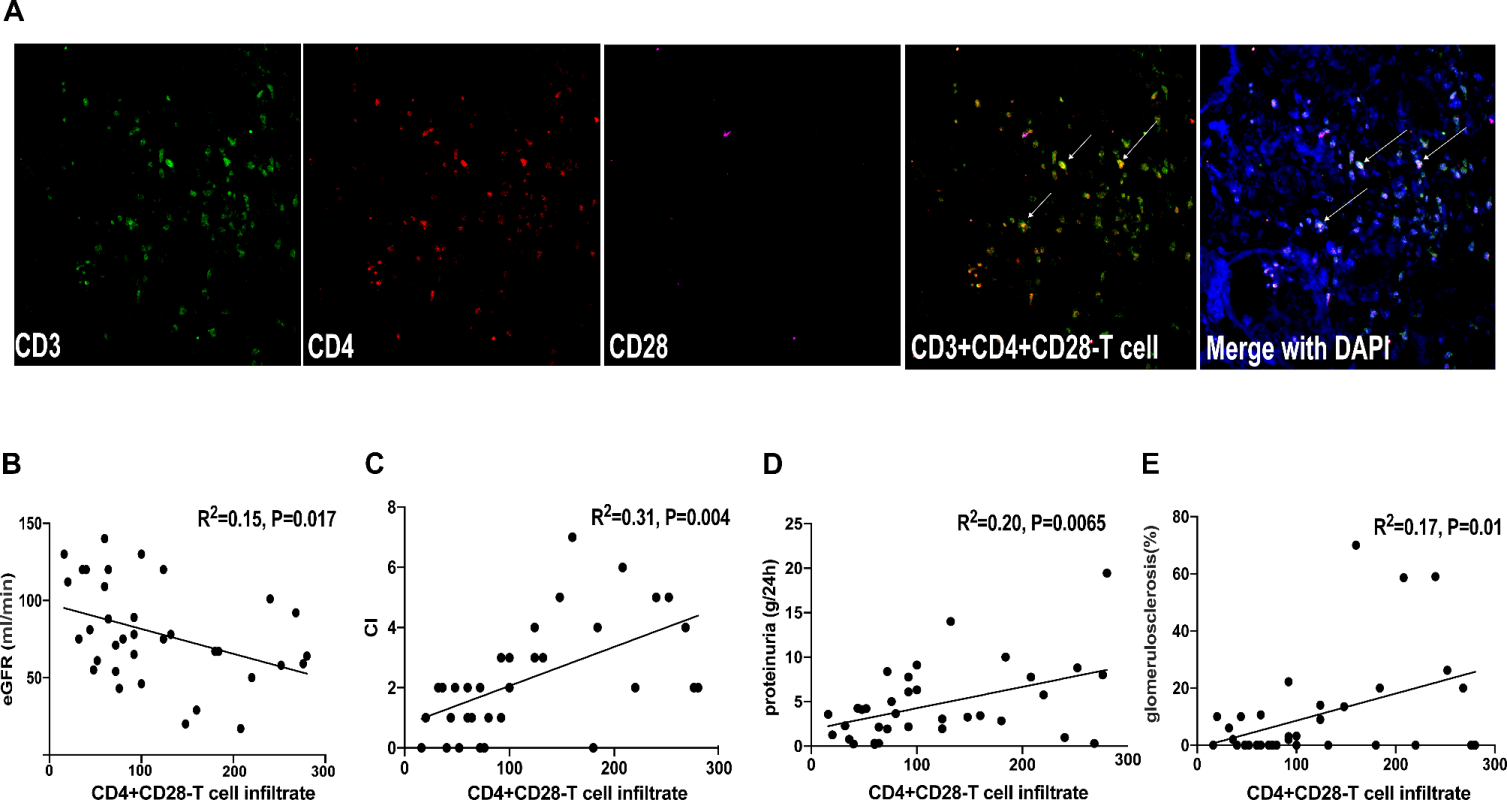
**CD4 + CD28− T cell filtration is correlated with the clinicopathological characteristics of LN patients** (A) Staining for CD3, CD4 and CD28 performed on SLE kidney slides showed that most infiltrating CD4 + T cells did not express CD28. Arrows point to CD3 + CD4 + CD28−T cells (B-E) Correlation between the CD4 + CD28− T cell population and clinicopathological characteristics in 35 LN IV patients: B, negative correlation with GFR; C-E, positive correlation with chronicity index, proteinuria and glomerulosclerosis

CD3, CD4 and CD28 immunostaining were used to examine CD4 + CD28−T cell infiltration in the renal tissues from the 35 patients diagnosed with proliferative LN and 3 healthy controls. As shown in Fig. 2A, CD4 + CD28−T cells infiltrated in the interstitial and periglomerular tissues, while most infiltrating CD4 + T cells from renal biopsy were negative for CD28. However, no CD4 + T-cell infiltration was detected in the normal renal tissue from the patients who underwent total kidney removal for other medical reasons (their normal renal tissues were used as healthy controls) (**Supplementary Fig.** 3). In each biopsy specimen, at least 10 glomeruli were examined. The correlation between the clinicopathological parameters of the renal lesions and the quantity of infiltrating CD4 + CD28− T cells per mm^2^ was further evaluated. Surprisingly, the number of infiltrating CD4 + CD28− T cells was negatively correlated with the estimated glomerular filtration rate (eGFR), but positively correlated with proteinuria, the proportion of glomerulosclerosis and the degree of chronicity of the LN renal tissue (Fig. 2B-E). These results indicate that CD4 + CD28− T cells were associated with the local renal damage and prognosis of LN.

### IL-15 level in the circulation and renal tissue of LN patients is significantly higher than that in healthy controls

**Fig. 3:**
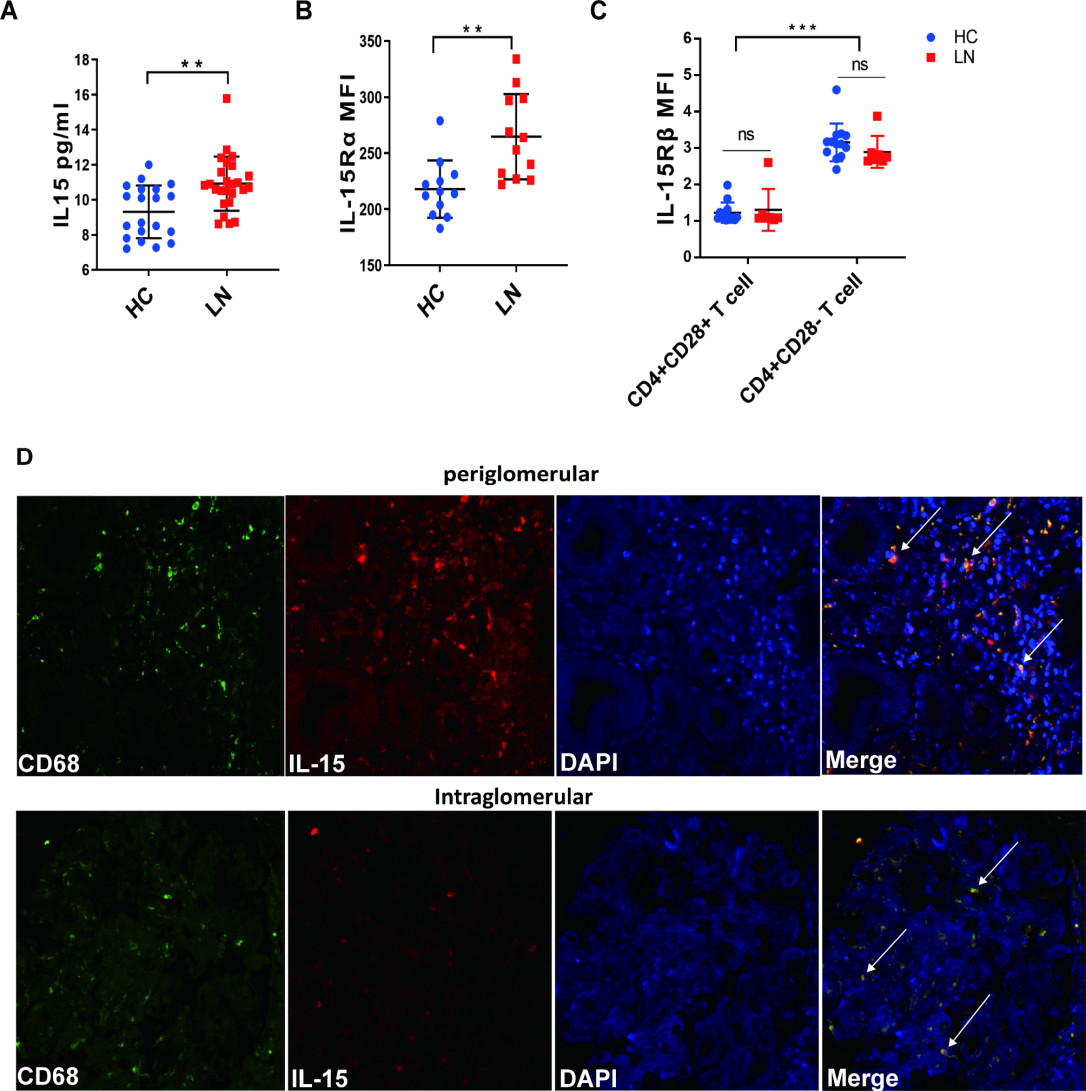
**IL-15 level is higher in the circulation and renal tissue of LN patients than that in healthy controls** (A) Soluble IL-15 levels in the sera of 20 healthy controls and 25 LN patients; each circle represents one individual and horizontal bars indicate the mean (B) Mean ± SEM expression of IL-15Rα in CD14 + monocytes from 15 healthy controls and 18 LN patients (C) Mean ± SEM expression of IL-15 receptor (IL-15Rβ) on CD4 + CD28− and CD4 + CD28 + T cell cells in 15 healthy controls and 18 LN patients (D) Immunofluorescence showed that CD68 + macrophages (green) expressed IL-15 (red) in intraglomerular and periglomerular areas of LN. Arrows point to IL-15 expressing macrophages (yellow) **P < 0.01

To evaluate the role of IL-15-mediated pathology, the level of soluble IL-15 was measured in the sera of the controls and age- and gender-matched patients. The concentration of soluble IL-15 in LN patients was significantly higher than that in the healthy controls (P = 0.0011) (Fig. 3A). In addition, comparison of IL-15Rα expression in CD14 + monocytes between LN patients and healthy controls showed that IL-15Rα expression in CD14 + monocyte in LN patients was significantly higher than that in healthy controls (P = 0.0019) (Fig. 3B). IL-15 functioned mainly in a cell contact-dependent manner through the trans-presentation of membrane-bound IL15–IL-15Rα complexes to respond to cells that expressed IL-2/IL-15Rβ–γ. IL15Rβ expression level in CD4 + CD28− T cells was significantly higher than that in CD4 + CD28 + T cells in LN patients, suggesting that CD4 + CD28− T cells had a greater advantage in response to IL-15 compared with CD4 + CD28 + T cells (Fig. 3C). To further evaluate the localization of IL-15 in the kidney tissue, IL-15-expressing macrophages in the kidney were studied by immunostaining. It was found that CD68-positive macrophages expressed IL-15 in both periglomerular and intraglomerular tissues (Fig. 3D). In contrast, no IL-15-expressing macrophage infiltration was observed in the renal tissue from the healthy controls (**Supplementary Fig.** 4).

### IL-15 enhances the cytotoxic function and cytokine secretion of CD4 + CD28- T cells

**Fig. 4:**
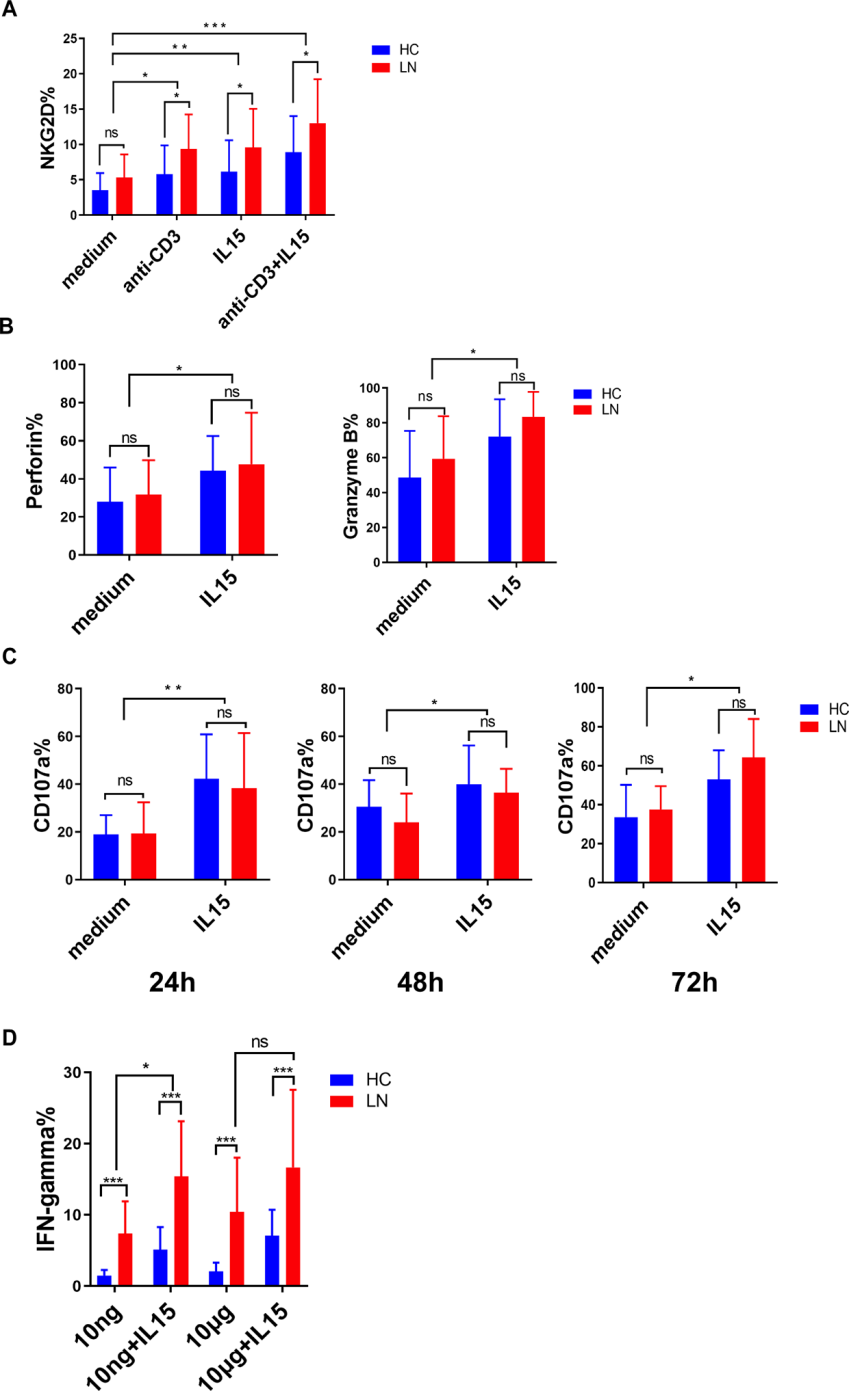
**IL-15 enhances innate function and cytokine secretion of CD4 + CD28− T cells** (A) PBMCs of 8 healthy controls and 8 LN patients were stimulated with IL-15 (50ng/ml), anti-CD3 (10ug/ml), IL-15 (50ng/ml) combined with anti-CD3 (10ug/ml) or left unstimulated for 24 h and performed by flow cytometry. Expression levels of NKG2D in CD4 + CD28−T cells are shown for healthy controls and LN patients. Graphs represent mean values of all donors (B) PBMCs of 8 healthy controls and 8 LN patients were stimulated with IL-15 (50ng/ml), or left unstimulated for 72 h and examined by flow cytometry. Percentages of perforin and granzyme B expression in CD4 + CD28−T cells are shown for healthy controls and LN patients. Graphs represent mean values of all donors (C) PBMCs of 8 healthy controls and 8 LN patients were stimulated with anti-CD3 (10ug/ml) with or without IL-15 (50ng/ml) for 72d. The frequency of degranulating (CD107a+) cells was examined every 24 h by flow cytometry. Percentages of degranulating (CD107a+) cells are shown for healthy controls and LN patients (D) PBMCs of 8 HCs and 8 LN patients were stimulated with IL-15 (50ng/ml) combined with anti-CD3 10ng/ml or 10ug/ml for 6 h and IFN -γ production was measured. The mean ± SEM production of IFN -γ of CD4 + CD28− T cells is shown. *p < 0.05, **p < 0.01, ***p < 0.001

Compared with CD4 + CD28 + T cells, CD4 + CD28−T cells exhibited cytotoxic properties including higher expression of NKG2D, perforin, granzyme B and stronger degranulation. Further detection of changes in the expression of these innate function-associated molecules of CD4 + CD28− T cells cultured with IL-15 showed that the expression level of NKG2D in CD4 + CD28− T cells from LN patients was higher than that from the healthy controls when cells were cultured with anti- CD3, IL-15 or both (Fig. 4A). After 72 h treatment with IL-15, the expression of perforin and granzyme B was increased markedly in CD4 + CD28− T cells from LN patients (Fig. 4B). Degranulation was provoked by anti-CD3 stimulation in the presence or absence of IL-15 for 24, 48 or 72 h as shown by the expression of the degranulation marker CD107a. It was found that CD107a expression was higher after combined stimulation of CD3 and IL-15 than that after CD3 stimulation alone at all time points **(**Fig. 4C**).**

We then checked IFN gamma production of CD4 + CD28-−T cells in LN patients and healthy controls. After stimulation of PBMCs with optimal 10ug/ml anti-CD3 or suboptimal 10ng/ml anti-CD3 with or without IL15, intracellular IFN -γ was quantified on CD4 + CD28−T cells. There was no significant difference in IFN -γ production in CD4 + CD28−T cells between optimal CD3 stimulation and suboptimal stimulation, further demonstrating that the activation of CD4 + CD28−T cells was not highly dependent on TCR ligation (Fig. 4D). However, IL15 only could augment IFN -γ production of LN CD4 + CD28−T cells under suboptimal stimulation, while the effect was not observed under optimal stimulation in LN patients (Fig. 4D). All these results suggested that CD4 + CD28−T cells in LN patients could produce higher levels of IFN -γ as compared with those in healthy controls under the above conditions.

### IL-15 selectively promotes the migratory potential of CD4 + CD28− T cells

**Fig. 5:**
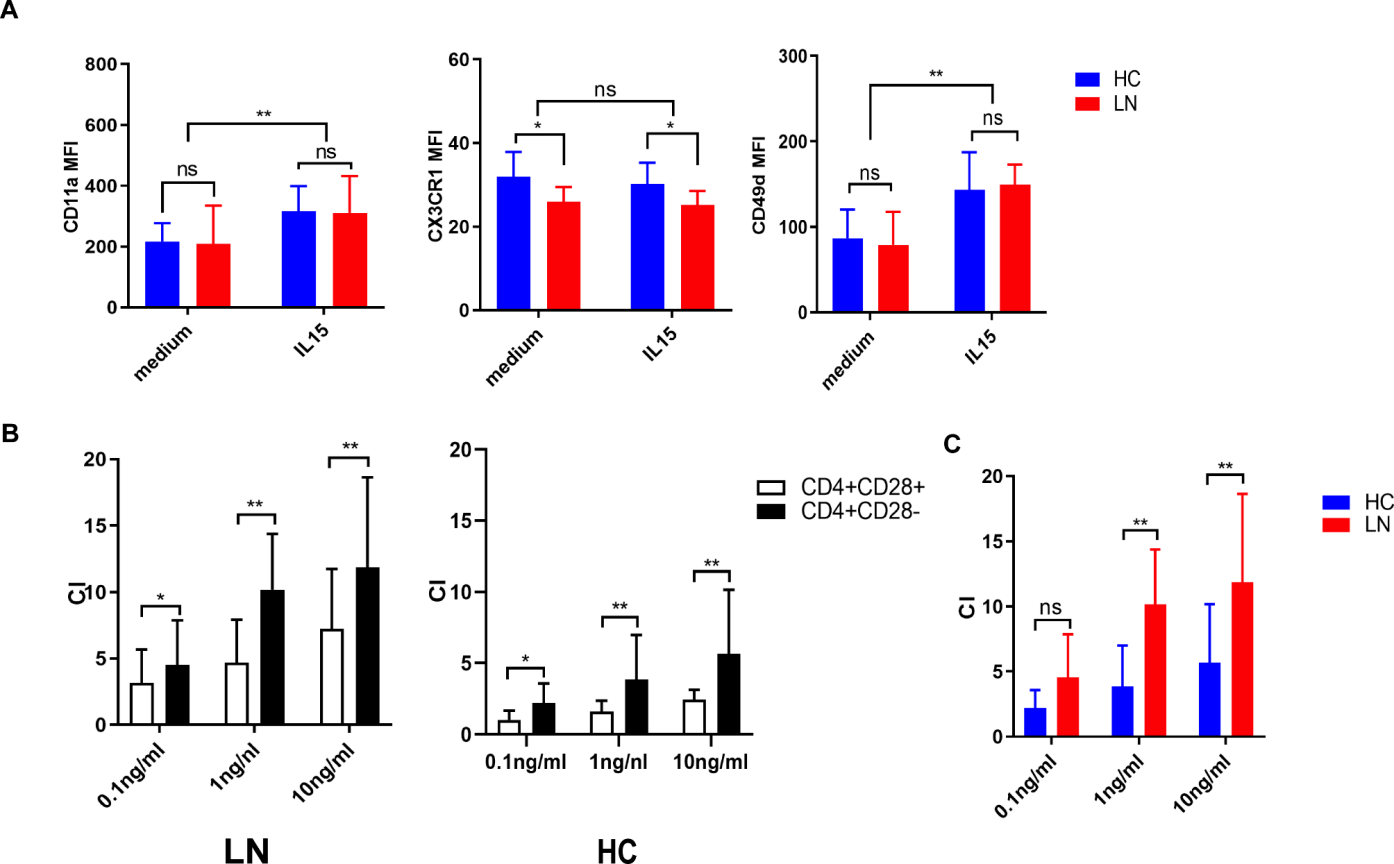
**IL-15 promotes migration potential of CD4 + CD28− T cells** (A) PBMCs of 8 healthy controls and 8 LN patients were stimulated with IL-15 (50ng/ml) or left untreated for 48-72 h and expression levels of CD11a, CD49d and CX3CR1 were measured by flow cytometry. The MFI of each marker within CD4 + CD28−T cells is shown (B) Sorted CD4 + T cells of 8 healthy controls and 8 LN patients were allowed to migrate in a transwell system toward different concentrations of IL-15 (0.1–10ng/ml). The number of migrated CD4 + CD28 + and CD4 + CD28− T cells was checked by flow cytometry. The chemotactic index (CI) was calculated as follows: (number of migrated cells with IL-15)/(number of migrated cells without IL-15) as described by Bieke Broux etc. CI of CD4 + CD28− T cells was compared with CI of CD4 + CD28 + T cells both in LN and HC (C)Within CD4 + CD28− T cells, CI of SLE patients were compared with that of HCs. *p < 0.05, **p < 0.01, ***p < 0.001, ns means no significant

The expression levels of integrins CD11a, CD49d, and the chemokine receptor CX3CR1 after IL-15 treatment for 48-72 h were also detected by flow cytometry. The results showed that the expression of CD11a and CD49d was increased significantly in CD4 + CD28− T cells in both LN patients and healthy controls (Fig. 5A), while the expression of the chemokine receptor CX3CR1 remained significantly unchanged. In addition, the extent to migration of CD4 + CD28− T cells towards different concentrations of IL-15 was detected by transwell migration assay using the method described by Broux et al [18]. The result showed that CD28- T cells from both LN patients and healthy controls had a significant migration advantage over the respective CD4 + CD28 + T cells at all IL-15 concentrations tested. But with the concentration of IL-15 increasing, CD4 + CD28− T cells from LN patients exhibited a higher efficiency of migration than those from healthy controls (Fig. 5B, C).

### IL-15 enhances the pathological injurious effect of CD4 + CD28− T cells on human glomerular endothelial cells (GEnCs).

**Fig. 6:**
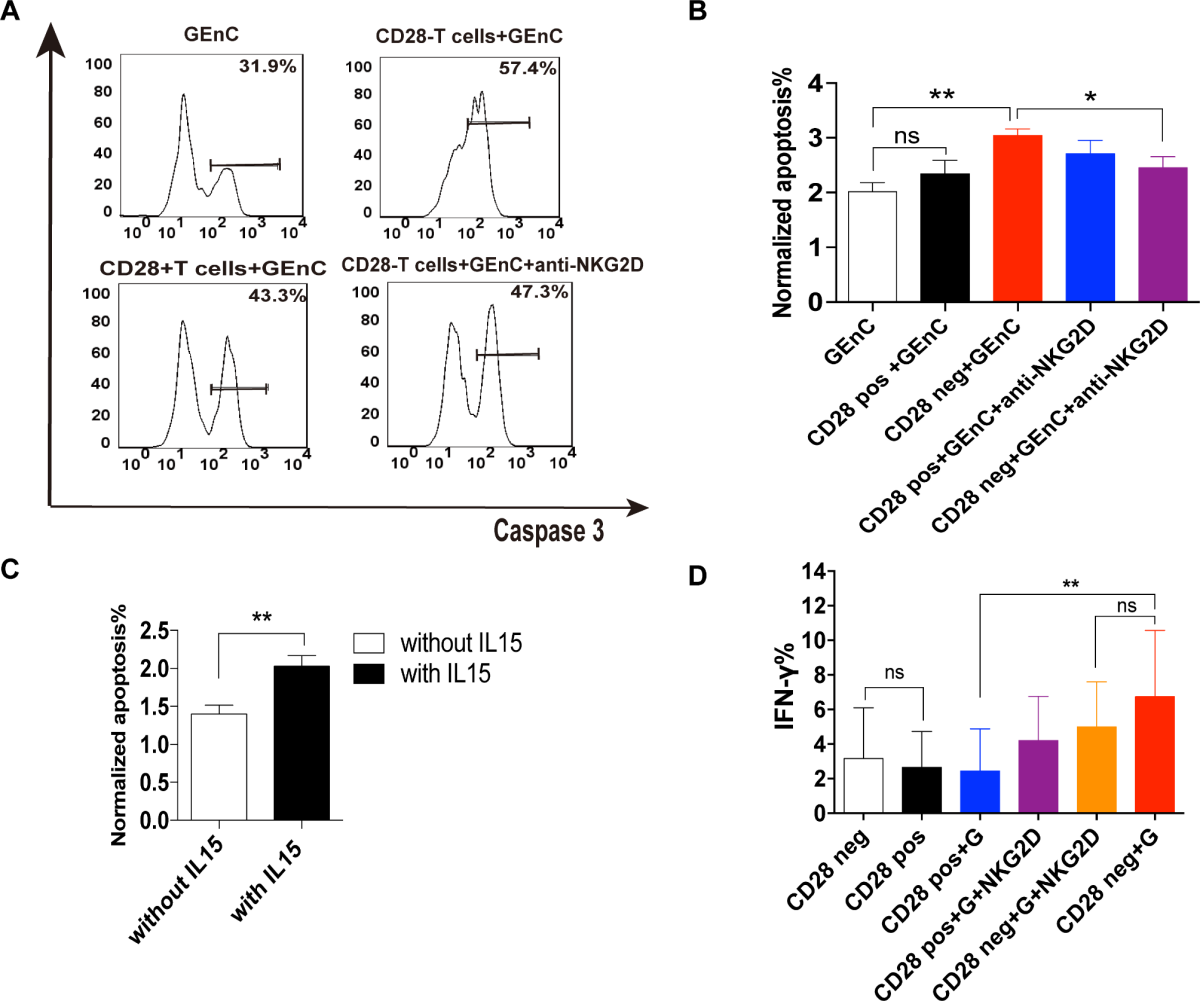
**CD4 + CD28− T cells induce higher apoptosis of GEnCs with IL-15 pre-treatment compared with** **CD4 + CD28 + T cells** After 16 h stimulation of 10^5^ CD4 + CD28 + or CD4 + CD28− T cells isolated from LN patients (n = 7) with IL-15 50ng/ml and 6h culture with GEnCs, the frequency of GEnCs expressing active caspase 3 and intracellular IFN-γ production in T cells were measured by flow cytometry (A) Expression of active caspase 3 of GEnCs (upper left), induced by CD4 + CD28 + T cells (bottom left), CD4 + CD28− T cells (upper right), the CD4 + CD28− T cells with anti-NKG2D blocking antibody (bottom right) (B) Data show the mean ± SEM normalized apoptosis rate of GEnCs in each condition (C) Data show the mean ± SEM normalized apoptosis rate of GEnCs without IL-15 treatment and with IL-15 treatment CD4 + CD28− T cells (D) Data show the mean ± SEM IFN -γ production of CD4 + CD28 + or CD4 + CD28− T cells in each condition. *p < 0.05, **p < 0.01, ns means no significance

As a higher number of CD4 + CD28− T cells and a higher level of IL-15 were observed in the kidney tissues of LN patients (Fig. 2A, 3D), we further evaluated the pathogenic function of LN CD4 + CD28− T cells under conditions of IL-15 stimulation by using GEnCs as target cells to monitor endothelial injury. The sorted CD4 + CD28− T cells and CD4 + CD28 + T cells from LN patients were pretreated with IL-15 for 16 h and then co-cultured with GEnCs in vitro to measure intracellular levels of IFN-γ and caspase3 in T cells and GEnCs. Strikingly, the CD4 + CD28− T cells induced a higher rate of GEnC apoptosis as compared with CD4 + CD28 + T cells (Fig. 6A, B). The results further showed that apoptosis was partially inhibited by an NKG2D-blocking antibody (P = 0.025) (Fig. 6A, B). Meanwhile, CD4 + CD28− T cells with IL-15 pre-stimulation induced a significantly higher apoptosis rate compared with CD4 + CD28− T cells without IL15 pre-stimulation (P = 0.0063) (Fig. 6C). Furthermore, CD4 + CD28− T cells displayed higher IFN-γ production than did the CD4 + CD28 + T cells after co-culturing with GEnCs, and the addition of the NKG2D-blocking antibody could not decrease this high level of IFN-γ production (Fig. 6D). CD4 + CD28−T cells derived from HC induced GEnC apoptosis rate was also studied. The CD4 + CD28−T cells from healthy control (HC) displayed a similar induced apoptosis pattern with that of LN.

## Discussion

Here we demonstrate that proinflammatory cytokine IL-15 enhanced the pathogenic nature of CD4 + CD28− T cells in LN in vitro. Although the local site of injury could not be fully presented with fidelity by studying T cells in the systemic level, we may provide new insights into the mechanism of CD4 + CD28− T cells related lupus tissue damage.

Previous studies showed that the shortened telomere length did not appear to reflect the disease activity [6]. Also, we also did not find a significant association between SLEDAI and the population of CD4 + CD28− T cells. Instead, our study observed a correlation between the SLE duration and CD4 + CD28− T cells, supporting that CD4 + CD28− T cells originate from repeated stimulations of auto-antigens or nonspecific stimuli from chronic inflammation [14, 21]. Besides, the expansion of CD4 + CD28− T cells was also found to be associated with CMV infection [22]. However, according to an epidemiological study conducted in China, the CMV seroprevalence was 98.11% in 6729 pregnant women underwent CMV serological testing in early gestation [23]. Therefore, the high population of CD4 + CD28 + T cell is suggested to be associated with premature aging in SLE patients.

It was found in our study that high expressions of NK cell receptors and cytotoxic cytokines could confer an innate-like function to CD4 + CD28− T cells of SLE patients. Additionally, we found that high expression of the integrins CD11a and CD49d and the chemokine receptor CX3CR1 facilitated the infiltration of CD4 + CD28− T cells into the renal tissue. Indeed, CD4 + CD28− T cell infiltration was confined to periglomerular or tubular epithelia in LN, indicating that the cells had formed an immunological synapse with their target cells [24]. Our previous study also revealed that CD4 + T infiltration in the tubulointerstitium was best correlated with chronicity of LN pathological lesions, which is known to be related to progressive renal failure [25]. Accumulating evidence shows that immunosenescence is accelerated in patients with chronic kidney disease [26]. A reduction in CD28 expression both in CD4 and CD8 subpopulations was also found in patients with end stage renal disease [27]. Furthermore, CD4 + CD28− T cells were reported to be associated with endothelial dysfunction and arterial stiffness, which play a major role in the development of LN [28]. Our study suggests increased CD4 + CD28−T cells caused by SLE further exacerbates injury to the major organs, and forms a positive feedback; reducing CD4 + CD28−T cells in the circulation and tissue may therefore break this vicious cycle of positive feedback.

Our study suggests that IL-15 may be a key element to boost the cytotoxic function of CD4 + CD28− T cells and related tissue damage. The NKG2D expression in CD4 + CD28− T cells was enhanced by IL-15 stimulation. IL-15 also increased the expression of the integrins CD11a and CD49d, which could promote the recruitment and migratory capabilities of CD4 + CD28− T cells. Our transwell assay demonstrated that CD4 + CD28- T cells from LN patients had higher migratory potentials in the presence of increasing concentrations of IL-15 as compared with those from healthy controls, indicating that CD4 + CD28− T cells from LN patients have a relatively higher sensitivity and stronger migratory potential towards IL-15. Furthermore, tissue damage is also highly associated with IL-15. Detection of macrophage infiltration in nephritic kidneys at repeat biopsy usually indicates a poor diagnosis. A recent study reported that elevation of urinary IL-15 was a specific indicator for identifying active LN patients [20, 29]. We postulate that renal macrophages present CD4 + CD28− T cells, which promote their activation, adhesion and cytokine production in the renal tissue [30, 31].

To the best of our knowledge, this is the first study that used primary human GEnCs as the targets of CD4 + CD28− T cells. Our data showed that CD4 + CD28- T cells released higher levels of the proinflammatory cytokine IFN-γ and induced higher apoptosis after IL-15 pre-treatment as compare with CD4 + CD28− T cells receiving no pretreatment. However, there was no significant difference between CD4 + CD28− T cells and CD4 + CD28 + T cells in terms of their intracellular levels of IFN-γ and caspase3 in the absence of IL-15 pretreatment (Supplementary figure 5). GEnC injury is one of the main contributor of glomerular lesions, which is the feature of renal ageing [32, 33]. We postulate that blocking IL-15 may be able to potentially reduce the renal aging caused by the additional injury mediated by CD4 + CD28− T cells in LN patients.

In conclusion, our study demonstrated that excessive IL-15 was able to promote cytotoxic CD4 + CD28− T cell-mediated injury in GEnCs in LN. This finding may provide new insights into the mechanism underlying the premature aging of the renal tissue in LN.

## Methods

### Study subjects

Altogether 92 SLE patients with or without nephritis were recruited from the Department of Rheumatology and Immunology at Shanghai Changzheng Hospital and Changhai Hospital (Shanghai, China). Thirty-six age-matched healthy blood donors recruited from Shanghai Blood Transfusion Service Center as healthy controls (see Table 1 for clinical characteristics) and 35 renal biopsy tissues recruited from Nanjing Glomerulonephritis Registry were used to determine the correlations between infiltrating CD4 + CD28−T cells and clinicopathological features. All blood samples were collected at the time of hospital admission. Informed consent was obtained from both patients and controls before initiation of the study. Complete details of the entire study design and procedures involved were in accordance with the Declaration of Helsinki. The study protocol was approved by the Ethics Committee of Shanghai Changzheng Hospital.

**Table 1:** Clinical and demographic characteristics of SLE/LN patients and healthy controls

Category	Features	SLE without nephritis (n = 10)	SLE with nephritis (n = 82)	Healthy donors(n = 36)
Demographic	Age (mean ± SD)	38.7 ± 11.3	32.9 ± 12.0	34.9 ± 11.2
	Female (%)	100	96.2	100
	Asian (%)	100	100	100
Disease activity	SLEDAI (mean ± SD)	8.27 ± 4.10	10.26 ± 4.36	
	Anti-dsDNA antibody (n, %)	6 (54.5)	39 (48.1)	
	C3 complement (g/L, mean ± SD)	0.44 ± 0.20	0.53 ± 0.26	
Medication	Naive (n, %)	7 (63.6)	44 (54.3)	
	Prednisone, currently taking (n, %)	4 (36.3)	37 (45.6)	
	Hydroxychloroquine (%)	4 (36.3)	30 (37.0)	
	Mycophenolate Mofetil exposure (%)	2 (18.1)	12 (14.8)	
	Cyclophosphamide exposure (%)	1 (9.1)	16 (12.5)	

SLE, systemic lupus erythematosus; LN, lupus nephritis

### Flow cytometry

Peripheral blood mononuclear cells (PBMCs) were isolated from EDTA-anticoagulated peripheral blood by centrifugation on Ficoll-Hypaque gradients (Sigma-Aldrich). For surface staining, antibodies specific for CD4 (labelled with FITC or Pacific Blue), CD28 (PE or APC), NKG2D (PE), CD11a (PE), CD62L (PECY7), CX3CR1 (APC) and CD49d (PE) were purchased from Biolegend (San Diego, USA). For characterization of adhesion molecules, integrins and chemokine molecules, PBMCs from the healthy controls and LN patients were seeded in 96-well round-bottom plates at 1 × 10^6^ cells/well in culture medium and stimulated with 50ng/ml recombinant human IL-15 (R&D Systems, Minneapolis, USA). After 48-72 h culture, cells were stained with the corresponding antibodies. PBMCs were stimulated with anti-CD3 (1µg/ml), IL-15 (50ng/ml) or both, and cultured for 24 h for NK receptor analysis and 72 h for cytotoxic molecule analysis. Cells were surface stained or intracellularly stained with anti-NKG2D-PE, anti-perforin-PE, anti-granzyme B-APC and anti-IFN-gamma-PE. All samples were analyzed by flow cytometry using an LSR II (BD Biosciences) or a Cyan flow cytometer (Beckman, CA, USA).

### Degranulation assay

PBMCs were cultured alone or stimulated with 50 ng/ml IL-15 for 3 days. The percentage of cells that degranulated was measured daily for 3 days. Anti-human CD107a-PE antibody, Golgistop (both BD Biosciences), and 2 µg/ml anti-CD3 (OKT3; BD Biosciences) were added 4 h before analysis. Flow cytometry was performed by labelling cells with the anti-CD4-Pacific Blue and anti-CD28-APC (both Biolegend). Samples were analyzed by flow cytometry using a Cyan flow cytometer (Beckman Coulter).

### Chemotaxis assay

PBMCs from the healthy controls and SLE patients were sorted by negative selection using the CD4 + T Cell Isolation Kit II (Miltenyi Biotech). A transwell system with a pore size of 0.5 mm (Corning, Lowell, MA) was used for migration assay. In the bottom compartment, IL-15 (R&D Systems) was added at increasing concentrations of 0.1ng/ml, 1ng/ml and 10 ng/ml. 5 × 10^5^ CD4 + T cells were seeded in each insert. After 5 h incubation, the total number of migrating cells was counted on a Cyan flow cytometer (Beckman Coulter), and the percentage of both CD4 + CD28− T and CD4 + CD28 + T cells was determined. The chemotactic index (CI) was calculated as previously described [18], by dividing the number of cells that migrated in the presence of IL-15 by the number of cells that migrated in the absence of IL-15.

### Measurement of impairment to GEnCs

GEnCs were purchased from Siencell (CA, USA). CD28− and CD28 + T cells were sorted from purified CD4 + T cells using the CD28 Microbead kit II (Miltenyi Biotech, Kölle, Germany). The purity of the sorted CD28- and CD28 + T cells ranged from 90 to 95%. Cells were stimulated with IL-15 (50ng/ml) for 24 h and then incubated with GEnCs in 96-well plates for 6 h. One well per patient was incubated with either 1 µg/ml anti-NKG2D antibody or an isotype control (BD Biosciences). Cells were stained for activated caspase 3 (as a marker of apoptosis) using anti-caspase 3-PE (BD Biosciences) and also stained with anti-IFN-gamma-Pacific Blue following staining for surface markers using anti-CD31-APC (GEnC) and anti-CD28-PE (Biolegend). Cell apoptosis rates and IFN-γ secretion were analyzed by flow cytometry using a Cyan flow cytometer (Beckman Coulter).

### Renal histopathology and immunostaining

All biopsies of the 35 patients with LN IV underwent light, immunofluorescence and electron microscopy for primary diagnosis and categorized based on the ISN/RPS classification by two pathologists independently. The activity index and chronicity index were determined using the modified NIH system [34]. The glomerular and tubulointerstitial lesions were shown as the rate involved of all observed glomeruli or cortex respectively. Renal sections were blocked with PBS containing 10% appropriate serum and sequentially incubated overnight with the mix of primary antibodies (rabbit anti-human CD3; R&D Systems, mouse anti-human CD4; R&D Systems, goat anti-human CD28; R&D Systems) for staining CD3 + CD4 + CD28− T cells. Secondary antibodies included donkey anti-rabbit Alexa Fluor 546; Invitrogen, donkey anti-mouse Alexa Fluor 647; Invitrogen and donkey anti-goat Alexa Fluor 488; Invitrogen. For staining IL-15-expressing macrophage, sections were incubated with primary antibodies (mouse anti-human CD68; R&D Systems, gout anti-human IL-15; R&D Systems). Secondary antibodies were donkey anti-mouse Alexa Fluor 488; Invitrogen and donkey anti-gout Alexa Fluor 594; Invitrogen. The slides were washed thoroughly in TBS three times and dried naturally before analysis. The stained tissues were evaluated by counting cells per mm^2^ using computer-assisted image analysis software (Count, Biomas, Erlangen, Germany) or by using a grid. Images were taken using confocal microscope imaging (Zeiss LSM900, Germany) or a DS-2 M-BW-C 2-megapixel camera.

## Statistical analysis

The results are expressed as the mean values ± SEM. Pearson’s correlation analysis was used to measure correlation between two parameters. Continuous data were analyzed by using the unpaired t test or the Mann–Whitney test, as appropriate. A two-tailed p value < 0.05 was considered statistically significant. All statistical analyses were performed using SPSS 25 software (IBM Corp., Armonk, New York, USA), and figures were plotted using GraphPad Prism 7.0 (GraphPad Software Inc. La Jolla, CA, USA).

## Electronic supplementary material

Below is the link to the electronic supplementary material.

Supplementary Material 1

## Data Availability

The corresponding author will provide the data used in this study upon request.
