# Supplementary material for: Excessive IL-15 promotes cytotoxic CD4 + CD28− T cell-mediated renal injury in lupus nephritis
Source: Immun Ageing. 2022 Nov 1;19:50. doi: 10.1186/s12979-022-00305-9 (PMC9624042; doi:10.1186/s12979-022-00305-9)
Supplement: Supplementary file 1 — Supplementary Material 1 [file 12979_2022_305_MOESM1_ESM.pdf]

Supplementary Fig.S1

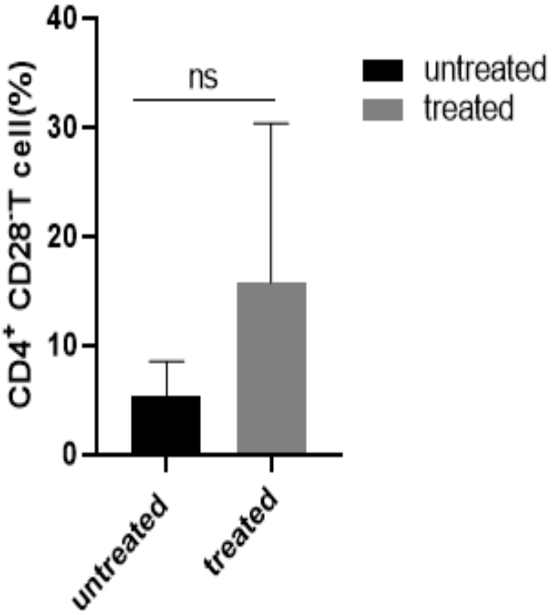

Supplementary Fig.S2

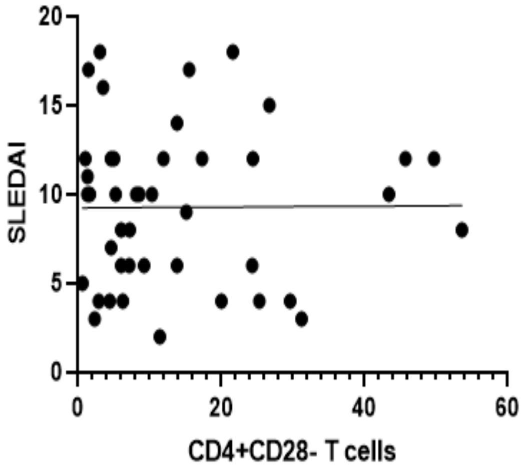

Supplementary Fig.S3

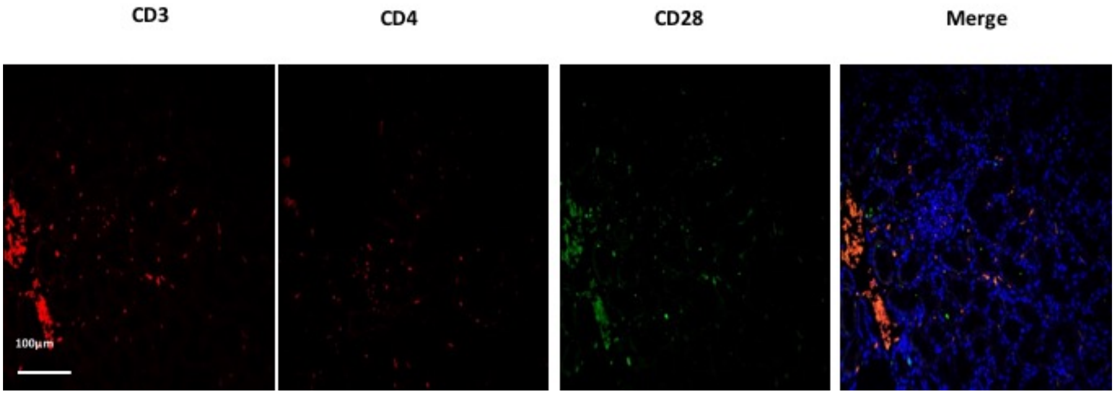

Supplementary Fig.S4

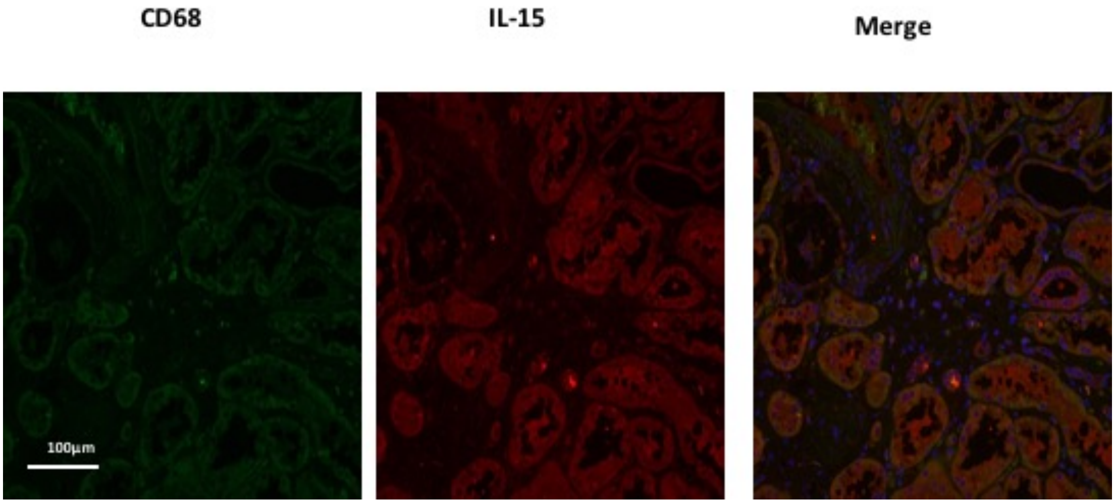

Supplementary Fig.S5

A **B**

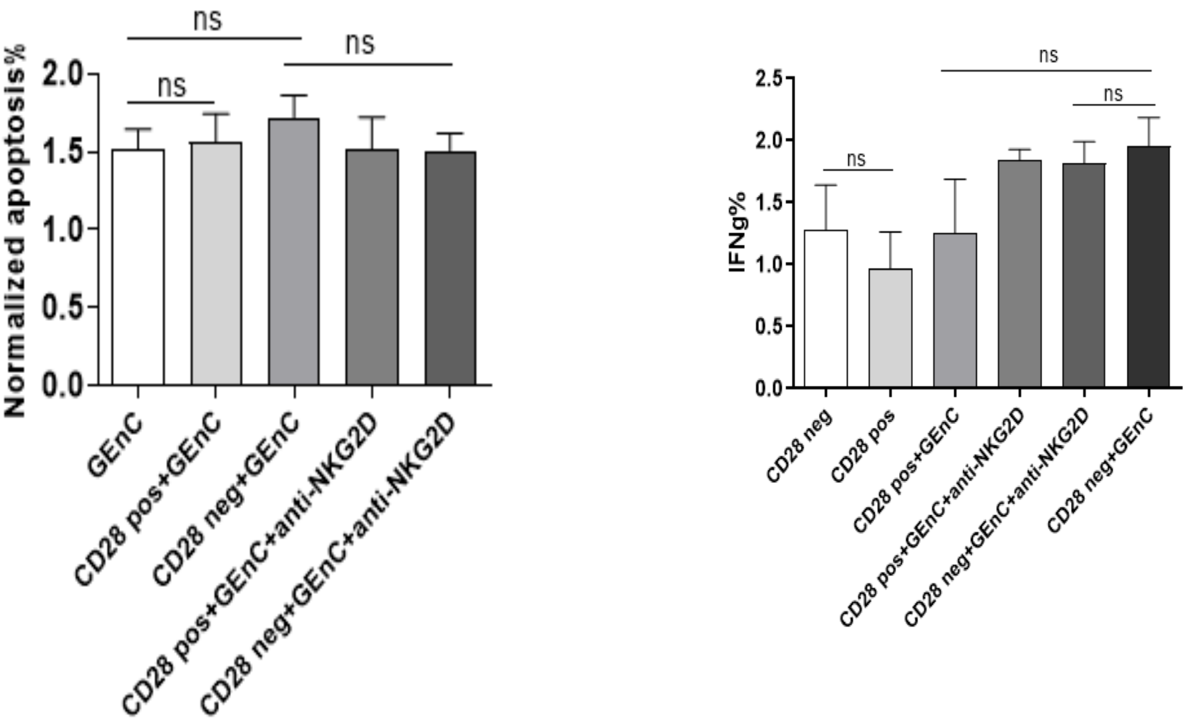

Figure S1 No significant difference of CD4+CD28-T cell population between treated and untreated SLE patients. CD4+CD28-T cell population from 7 untreated SLE patients and 36 treated SLE patients were compared. ns means not significant.

Figure S2 No significant correlation between CD4+CD28-T cell population and SLEDAI scores in SLE patients. The correlation between SLEDAI scores and CD4+CD28-T cell population from 43 SLE patients was determined by Pearson's correlation analysis.

Figure S3 No CD4+T cells infiltrated the kidney of HCs. Double staining for CD4 and CD28 was performed and showed no infiltrating CD4+ T cells in HC kidney slides. Original magnification x400

Figure S4 Double staining for CD68 and IL-15 performed on HC kidney slides. The data show that no macrophages infiltrated HC renal tissues. Original magnification x400

Figure S5 The normalized apoptosis rates of GEnCs, and the levels of IFN-gamma produced by CD4+CD28- T cells and CD4+CD28+ T cells that did not receive IL-15 pretreatment. 10^5 CD4+CD28+ or CD4+CD28- T cells isolated from patients with SLE (n=7) were incubated with GEnCs for 6 h, and the frequency of GEnCs expressing active caspase3 and the levels of intracellular IFN-gamma in T cells were measured by flow cytometry. The data showed the mean±SEM of the normalized apoptosis rates of GEnCs in each condition (A). The data showed the mean±SEM IFN-gamma production by CD4+CD28+ or CD4+CD28- T cells in each condition (B). ns means not significant.
